# Automatic COVID-19 Detection Using Exemplar Hybrid Deep Features with X-ray Images

**DOI:** 10.3390/ijerph18158052

**Published:** 2021-07-29

**Authors:** Prabal Datta Barua, Nadia Fareeda Muhammad Gowdh, Kartini Rahmat, Norlisah Ramli, Wei Lin Ng, Wai Yee Chan, Mutlu Kuluozturk, Sengul Dogan, Mehmet Baygin, Orhan Yaman, Turker Tuncer, Tao Wen, Kang Hao Cheong, U. Rajendra Acharya

**Affiliations:** 1 School of Management & Enterprise, University of Southern Queensland, Toowoomba 2550, Australia; Prabal.Barua@usq.edu.au; 2 Department of Biomedical Imaging, Faculty of Medicine, University of Malaya, Kuala Lumpur 50603, Malaysia; nadia_fareeda@ummc.edu.my (N.F.M.G.); kartini@ummc.edu.my (K.R.); norlisah@ummc.edu.my (N.R.); wei.lin@ummc.edu.my (W.L.N.); waiyeec@ummc.edu.my (W.Y.C.); 3 Department of Pulmonology Clinic, Firat University Hospital, Firat University, Elazig 23119, Turkey; mutlukuluozturk@hotmail.com; 4 Department of Digital Forensics Engineering, College of Technology, Firat University, Elazig 23119, Turkey; orhanyaman@firat.edu.tr (O.Y.); turkertuncer@firat.edu.tr (T.T.); 5 Department of Computer Engineering, College of Engineering, Ardahan University, Ardahan 75000, Turkey; mehmetbaygin@ardahan.edu.tr; 6 Science, Mathematics and Technology Cluster, Singapore University of Technology and Design, 8 Somapah Road, Singapore S485998, Singapore; tao_wen@mymail.sutd.edu.sg; 7 Department of Electronics and Computer Engineering, Ngee Ann Polytechnic, Singapore S599489, Singapore; aru@np.edu.sg; 8 Department of Biomedical Engineering, School of Science and Technology, Singapore University of Social Sciences, Singapore S599494, Singapore; 9 Department of Biomedical Informatics and Medical Engineering, Asia University, Taichung 41354, Taiwan

**Keywords:** Exemplar COVID-19FclNet9, deep feature generation, transfer learning, COVID-19 detection, iterative NCA

## Abstract

COVID-19 and pneumonia detection using medical images is a topic of immense interest in medical and healthcare research. Various advanced medical imaging and machine learning techniques have been presented to detect these respiratory disorders accurately. In this work, we have proposed a novel COVID-19 detection system using an exemplar and hybrid fused deep feature generator with X-ray images. The proposed Exemplar COVID-19FclNet9 comprises three basic steps: exemplar deep feature generation, iterative feature selection and classification. The novelty of this work is the feature extraction using three pre-trained convolutional neural networks (CNNs) in the presented feature extraction phase. The common aspects of these pre-trained CNNs are that they have three fully connected layers, and these networks are AlexNet, VGG16 and VGG19. The fully connected layer of these networks is used to generate deep features using an exemplar structure, and a nine-feature generation method is obtained. The loss values of these feature extractors are computed, and the best three extractors are selected. The features of the top three fully connected features are merged. An iterative selector is used to select the most informative features. The chosen features are classified using a support vector machine (SVM) classifier. The proposed COVID-19FclNet9 applied nine deep feature extraction methods by using three deep networks together. The most appropriate deep feature generation model selection and iterative feature selection have been employed to utilise their advantages together. By using these techniques, the image classification ability of the used three deep networks has been improved. The presented model is developed using four X-ray image corpora (DB1, DB2, DB3 and DB4) with two, three and four classes. The proposed Exemplar COVID-19FclNet9 achieved a classification accuracy of 97.60%, 89.96%, 98.84% and 99.64% using the SVM classifier with 10-fold cross-validation for four datasets, respectively. Our developed Exemplar COVID-19FclNet9 model has achieved high classification accuracy for all four databases and may be deployed for clinical application.

## 1. Introduction

The COVID-19 pandemic is an ongoing global pandemic caused by the severe acute respiratory syndrome coronavirus 2 (SARS-CoV-2) [1,2,3,4,5,6,7]. The pandemic has resulted in 3.89 million deaths worldwide, with 180 million confirmed cases thus far [8]. As in many other diseases, early detection of COVID-19 helps to provide timely treatment and save one’s life. The real-time reverse transcription polymerase chain reaction (RT-PCR) test is widely used to diagnose COVID-19 [9]. However, it can achieve erroneous results and has relatively long turnaround times. The test is also costly and deep nasal swabs can be uncomfortable for some people, especially small children. Therefore, relying only on the RT-PCR test may be inadequate for the diagnosis of COVID-19 under time-sensitive situations [10]. Clinical symptoms, laboratory findings, and radiological imaging techniques such as chest computed tomography (CT) or chest radiography (X-ray) images can also be used for screening. In particular, radiological imaging techniques play a significant role in the diagnosis of COVID-19 [11,12]. The diagnosis of COVID-19 is facilitated by the bilateral patchy shadows and ground-glass opacity obtained from these techniques [13,14]. Among these techniques, a chest X-ray is faster and cheaper than CT. It also causes lower-dose radiation on the patient compared to CT. Radiologists use these techniques to analyse images and help to diagnose COVID-19 [15,16].

Machine learning is a powerful technique used for automatic feature extraction [17,18,19,20]. Many machine learning techniques have been presented in the literature to detect different diseases [21,22,23,24,25]. Machine learning techniques developed especially for the early diagnosis of COVID-19 have achieved successful results [26,27]. For example, deep-learning-based methods from machine learning techniques are widely used for COVID-19 detection [28,29]. Deep-learning-based methods achieved high accuracy rates when sufficient labelled data is provided. Thus, deep-learning-based automatic diagnosis systems are of great interest in cases with no or few radiologists available [2]. Such an approach can also serve as an adjunct tool to be used by clinicians to confirm their findings.

### 1.1. Motivation and Our Method

COVID-19 is an infectious disease that has a relatively high infectivity rate. Many machine learning and signal/image processing methods have been employed to detect COVID-19 automatically using medical images or cough sounds. A new strategy is developed here to detect COVID-19 disease accurately using a novel Exemplar COVID-19FclNet9 model via both deep learning and feature engineering.

In this work, transfer learning is employed to generate hybrid deep features. It can be noted from feature engineering that exemplar feature generation is an effective method to extract discriminative features from an image. Exemplar feature extraction is processed using three pre-trained networks, namely AlexNet [30], VGG16 [31] and VGG19 [31]. These three networks are used as feature generators because each network has three fully connected layers. Using these fully connected layers, nine exemplar feature generation algorithms are then presented. The loss values of the generated features are calculated, and the top three feature vectors are selected to create a merged feature vector. The final feature vector is selected using an iterative selector; this selector is named iterative neighbourhood component analysis (INCA) [32], and the chosen feature vector is classified using a support vector machine (SVM) classifier [33,34]. Four different X-ray image databases have been utilised as testbeds to demonstrate the robustness of the presented model. We have utilised the advantages of the AlexNet, VGG16 and VGG19 CNNs together. The important features of the proposed Exemplar COVID-19FclNet9 are given below:

- choosing the most informative features automatically and
- denoting the effectiveness of this model using a conventional classifier. Bayesian optimisation is used to tune parameters of SVM classifier.

### 1.2. Literature Review

Different COVID-19 detection models have been presented in the literature. Narin et al. [35] proposed a COVID-19 detection model. This model was based on deep convolutional neural networks (Inception-ResNetV2, InceptionV3, ResNet50-101-152). ResNet50 was most accurate in their study compared to the other pre-trained models using an X-ray image dataset. Three different databases were used to automate the model. Database1 contained data from 341 COVID-19 and 2800 normal cases. Database2 consisted of data regarding 341 COVID-19 and 1493 viral pneumonia cases, and finally, database 3 had the data of 341 COVID-19 and 2772 bacterial images. Five-fold cross-validation was implemented to develop the model. In their study, accuracy rates of 96.10%, 99.50% and 99.70% were obtained using database1, database2 and database3, respectively. Muhammad and Hossain [36] presented a COVID-19 classification method using a convolutional neural network (CNN) with lung ultrasound images. They considered three classes in their work: COVID-19, pneumonia and healthy. They reported an accuracy rate of 92.50% with their proposed method. Loey et al. [37] applied a network based on CNN and generative adversarial networks. They used a database consisting of 307 chest X-ray images with 4 classes: COVID-19, viral pneumonia, bacterial pneumonia and normal, to develop the automated model. They achieved an accuracy of 80.60% with GoogleNet with four classes. Furthermore, they attained an accuracy rate of 100.0% for two classes (COVID-19 and normal). Their method did not achieve a high accuracy rate for four classes. Saad et al. [38] used GoogleNet, ResNet18 and deep feature concatenation for COVID-19 detection using CT and X-ray images. The data consisted of two classes: 2628 COVID-19 images and 1620 non-COVID-19 images. They achieved an accuracy of 96.13%. Moreover, with deep feature concatenation, accuracy rates were 98.90% and 99.30% with CT and X-ray databases, respectively. A high accuracy rate was obtained with this method for the two-class database. Tuncer et al. [39] proposed a COVID-19 detection method using a residual exemplar local binary pattern called ResExLBP. For this, images from 87 COVID-19 and 234 healthy patients were used in their study. They reported accuracy of 100.0% with SVM classifier. The main limitation of their method is that they used a small database to develop the model. Sharma and Dyreson [40] used a residual attention network for COVID-19 detection using chest X-ray images. In their study, 239 chest X-ray images were utilised. Their proposed method was compared with different CNN models. Their method attained an accuracy of 98.00%. Jia et al. [41] presented an approach based on CNN using CT and X-ray images. The modified MobileNet was used to classify five classes (COVID-19, tuberculosis, viral pneumonia, bacterial pneumonia and healthy). They obtained an accuracy of 98.80% for five-class classification. Bassi and Attux [42] proposed a deep CNN model to classify three classes (COVID-19, pneumonia and healthy) with 150 images. They reported an accuracy of 100.0%. Most of the works reported above have used smaller databases, or their proposed models are computationally intensive.

### 1.3. Contributions

The novelty of the Exemplar COVID-19FclNet9 model is in the presented exemplar deep feature extractor. The feature extraction model is designed as a machine learning model. The proposed fully connected layer–based feature generator contains deep exemplar feature extraction, feature selection based on NCA [43], misclassification rate calculation with SVM, feature vector selection using the calculated misclassification rates and concatenation steps. In this work, we present a machine learning model for feature generation, and this generator aims to use deep features with maximum effectiveness. The major contributions of the proposed Exemplar COVID-19FclNet9 are given below:

- This work presents a new X-ray image classification model using deep exemplar features. This model uses three cognitive phases, as described in Section 1.1. The proposed model is inspired by a Vision Transformer (ViT) [44]. In addition, this work presents a lightweight and highly accurate model using three pre-trained CNNs [30]. The proposed Exemplar COVID-19FclNet9 uses cognitive feature extraction, iterative feature selection and parameters to tune the SVM classifier to achieve high classification performance.
- Many machine learning models have been presented to classify COVID-19 [7,26,45]. The proposed Exemplar COVID-19FclNet9 model has been tested using four X-ray image databases. The universal high classification ability of the Exemplar COVID-19FclNet9 is used to justify the robustness of the developed model.

## 2. Materials and Methods

Details of four X-ray image databases (DB1, DB2, DB3 and DB4) used in this work are given in this section.

### 2.1. Materials

#### 2.1.1. The First Database (DB1)

**Figure 1:**
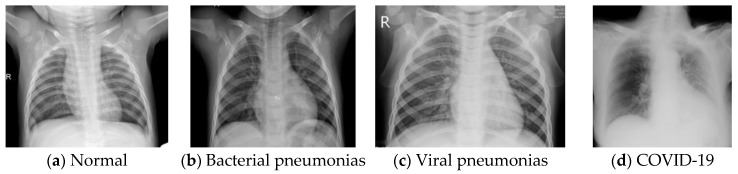
Sample of images from DB1.

The first database (DB1) used in this work consisted of 741 X-ray images with four classes (control/healthy, bacterial pneumonia, viral pneumonia and COVID-19). This database is a hybrid database in which normal and pneumonia images were taken from test images of Kermany et al.’s database [46,47]. COVID-19 images were taken from Talo’s database [48,49]. In DB1, we have used 234 normal, 242 bacterial pneumonia, 148 viral pneumonia, and 125 COVID-19 X-ray images. Typical images are shown in Figure 1.

#### 2.1.2. The Second Database (DB2)

**Figure 2:**
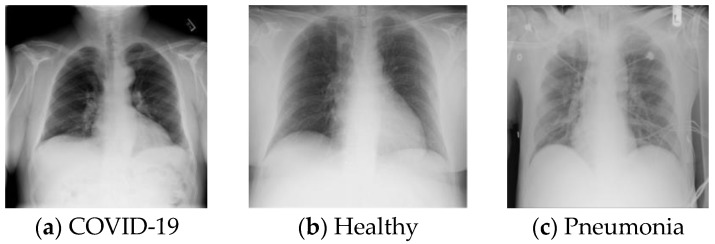
Sample of images from DB2.

This database is very popular [50] and was utilised to compare our results. Ozturk et al. [48] designed a novel machine learning model to detect COVID-19 and published their database and model in [49]. This database was collected from 125 subjects (43 females and 82 males). This database consists of three classes: COVID-19, pneumonia and control. The DB2 database contains 1125 (500 pneumonia, 500 control and 125 COVID-19) X-ray images. Typical images are shown in Figure 2.

#### 2.1.3. The Third Database (DB3)

**Figure 3:**
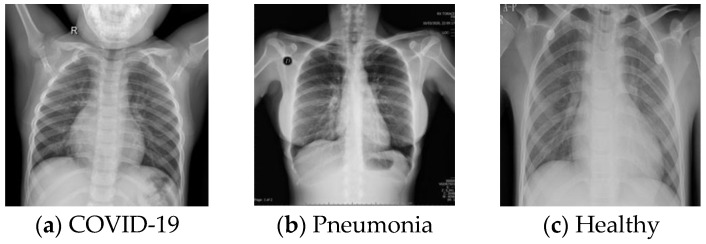
Sample of images from DB3.

This dataset is a large X-ray image dataset published by Rahman in Kaggle [51], which contains three classes: no-finding, pneumonia and COVID-19 [52,53]. We used 8961 X-ray images (3616 COVID-19, 1345 pneumonia and 4000 normal). Typical images are shown in Figure 3.

#### 2.1.4. The Fourth Database (DB4)

**Figure 4:**
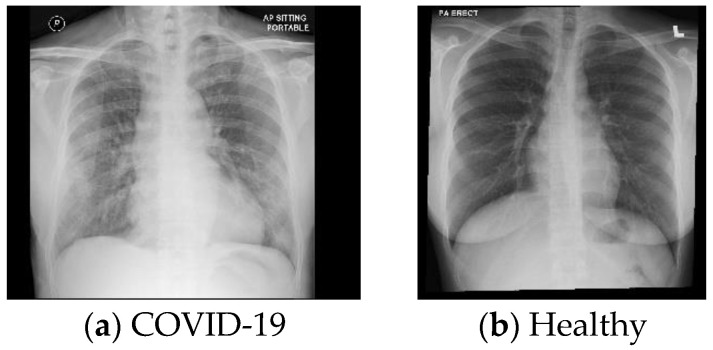
Sample of images from DB4.

This database was collected from the University of Malaya Medical Centre. In all, 277 X-ray images were collected from 214 subjects. This database consists of two categories, i.e., images from 127 COVID-19 and 150 healthy patients. Typical images are shown in Figure 4.

### 2.2. Methods

**Figure 5:**
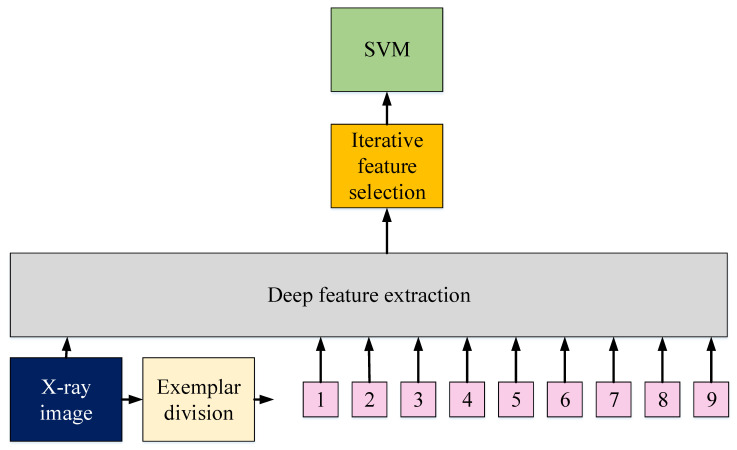
Graphical illustration of proposed Exemplar COVID-19FclNet9 model.

In this work, the Vision Transformer (ViT) [44] design is followed, and we modified this structure to propose our model using transfer learning. This research presents a hybrid model (it uses three pre-trained deep feature generators together) to achieve maximum classification ability, and it is named Exemplar COVID-19FclNet9. ViT inspires the proposed Exemplar COVID-19FclNet9, and it uses three pre-trained deep feature generators instead of attention transformers. The schematic overview of the proposed Exemplar COVID-19FclNet9 is denoted in Figure 5.

Figure 5 summarises the presented model. The pseudocode of the proposed Exemplar COVID-19FclNet9 X-ray classification model is given in Algorithm 1.
**Algorithm 1** The algorithm used to implement proposed Exemplar COVID-19FclNet9 model**Input:** X-ray image database**Output:** Results00: Load X-ray image database.01: **for** *k* = 1 to dim **do** // Herein, dim is number of images.02:    Read each image03:    Divide X-ray image into exemplars/patches04:    **for** *j* = 1 to 9 **do**05:      Generate deep features from X-ray images and patches using fully connected layers.06:      Merge generated features.07:      Create *j*th feature ($X^{j}$) vector of the *k*th.08:    **end for *j***09: **end for *k***10: **for** *j* = 1 to 9 **do**11:    Apply NCA to $X^{j}$ and calculate indexes (inx).12:    Select top 1000 features using inx.13:    Calculate misclassification rates of the chosen 1000 features.14: **end for *j***15: Select the best three chosen feature vectors.16: Merge the best three vectors.17: Employ iterative NCA to the merged features.18: Fed the chosen final feature vector to SVM classifier.19: Tune the parameters of the SVM classifier.20: Obtain results using the tuned SVM with 10-fold cross-validation.

More details about the proposed Exemplar COVID-19FclNet9 are given below.

#### 2.2.1. Deep Feature Extraction

**Figure 6:**
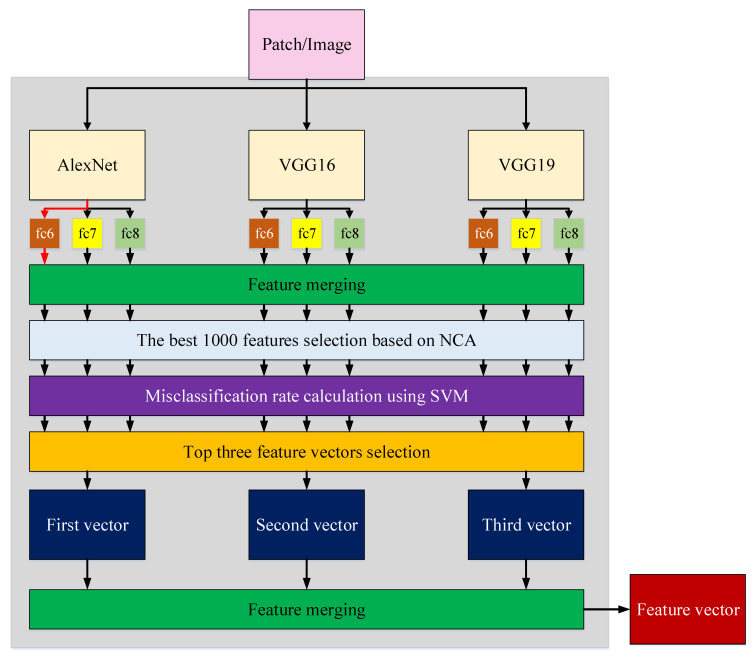
Detailed representation of proposed deep feature generator. The red arrow shows the activated feature extraction cell. All fully connected layers are activated consecutively in the presented hybrid deep feature generator.

Lines 01–16 of Algorithm 1 define the presented deep feature generator. In the first stage, exemplar division has been applied to the X-ray images. In this work, the X-ray images are divided into 3×3=9 exemplars. Then, in the deep feature generator, nine deep feature extractors (three fully connected layers of three pre-trained CNNs) have been applied to the obtained nine exemplars. Finally, the original X-ray images and the extracted features are merged. The schematic explanation of the presented deep feature generator is shown in Figure 6.

Steps of the presented deep feature generator are given below:

***Step 1:*** Create non-overlapping patches.

(1) $$pt^{cnt}\left(p,r,k\right)=I\left(i:i+p-1,\;j:j+r-1,k\right),cnt\in \left\{1,2,\ldots ,9\right\}$$

(2) $$p\in \left\{1,2,\ldots ,\lfloor \frac{w}{3}\rfloor \right\},r\in \left\{1,2,\ldots ,\lfloor \frac{h}{3}\rfloor \right\},i\in \left\{1,\lfloor \frac{w}{3}\rfloor ,\ldots ,w\right\},j\in \left\{1,\lfloor \frac{h}{3}\rfloor ,\ldots ,h\right\}$$

Equations (1) and (2) define patch creating. In this work, nine patches are created, where $pt^{cnt}$ is $cnt^{th}$ patch and cnt is a counter for the patches, I represents the original X-ray image, w denotes the width of the used X-ray image, h is the height of the used X-ray image and i,j,k,p,r are indices.

***Step 2:*** Extract nine features from X-ray images and patches using nine fully connected layers.

(3) $$X^{q}\left(h,1:sz\right)=fem^{q}\left(I\right),\;q\in \left\{1,2,\ldots ,9\right\},\;h\in \left\{1,2,\ldots ,dim\right\}$$

(4) $$X^{q}\left(h,q\times sz+1:\left(q+1\right)\times sz\right)=fem^{q}\left(pt^{cnt}\right)$$

In Equations (3) and (4), $X^{q}$ is *q*th feature vector, $fem^{q}$ is qth feature extraction method. In this work, fc6, fc7 and fc8 layers of the AlexNet, VGG16 and VGG19 were used to generate deep features, sz denotes the length of the generated feature vector. The fc8 layer of the used networks extracts 1000 features (sz is 1000 for $fem^{3},\;fem^{6},\;fem^{9}$). Moreover, 4096 features are generated using the fc6 and fc7 layers of all used CNNs (sz is 4096 for $fem^{1},\;fem^{2},\;fem^{4},\;fem^{5},\;fem^{7},\;fem^{8}$). The presented deep extractor creates features from X-ray images and nine patches. The Equations (3) and (4) define both feature generation and merging. The length of the generated $X^{3},\;X^{6},X^{9}$ is 10,000 and the length of the other feature vector is 40,960.

***Step 3:*** Select the best 1000 features from each generated feature vector deploying NCA.

(5) $$inx^{q}=NCA\left(X^{q},y\right)$$

(6) $$fc^{q}\left(h,j\right)=X^{q}\left(h,inx^{q}\left(j\right)\right),j\in \left\{1,2,\ldots ,1000\right\}\;$$

where $inx^{q}$ are the qualified indexes of the qth feature vector, y represents actual output, $NCA\left(.,.\right)$ defines NCA feature selection function and $fc^{q}$ is qth chosen feature vector with a length of 1000.

In this step, 1000 features are selected from generated feature vectors as in many pre-trained CNNs.

***Step 4:*** Calculate misclassification rates of the fc using polynomial kernelled SVM with 10-fold cross-validation.

(7) $$loss\left(q\right)=SVM\left(fc^{q}\right)$$

Herein, loss is the misclassification rate of the selected features.

***Step 5:*** Select the best three selected feature vectors using calculated loss values in Step 4.

(8) $$\left[ql,id\right]=sort\left(loss\right)$$

(9) $$tf^{i}=fc^{id\left(i\right)},i\in \left\{1,2,3\right\}\;$$

Herein, ql is qualified loss by ascending, $sort\left(.\right)$ defines the sorting function, id represents sorted indexes and $tf^{1},\;tf^{2},tf^{3}$ are the top three feature vectors.

***Step 6:*** Merge the selected three feature vectors and obtain generated features.

(10) $$X^{G}=tf^{1}\left|tf^{2}\right|tf^{3}$$

Herein, $X^{G}$ is the best-merged feature with a length of 3000 and | is the merging operator.

#### 2.2.2. Iterative Feature Selector

In order to choose the best features from the generated $X^{G}$, the iterative NCA (INCA) [32] feature selector was employed. INCA is an iterative and improved version of the NCA that helps to select the most appropriate feature vector with optimal length. It is a parametric feature selector, and the parameters of this selector are loss function and range of iteration (initial and end values). In this work, the SVM classifier was used as a loss function, and [100, 1000] was selected as the range. INCA can select different-sized feature vectors for different problems. The steps of the INCA are given below.

***Step 7:*** Generates qualified indexes of the features.

***Step 8:*** Select 901 features using generated qualified indexes. The lengths of the first and last feature vectors are 100 and 1000.

**Figure 7:**
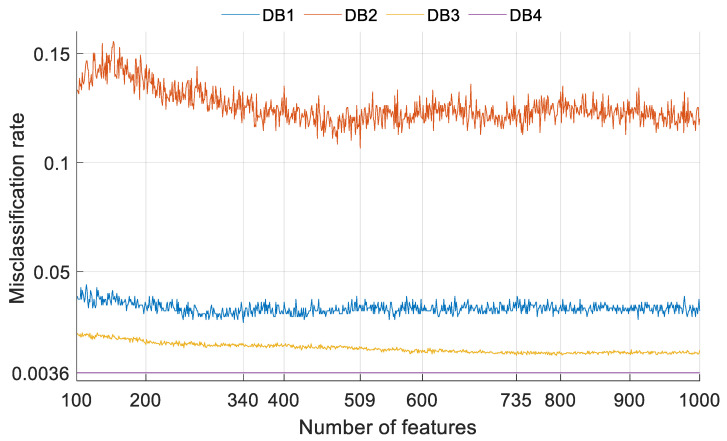
Plot of misclassification rate versus the number of features obtained for various databases.

***Step 9:*** Calculate loss values of each feature vector using SVM, which is equal to 1-accuracy (i.e., 901 in this case). The computed errors are shown in Figure 7.

***Step 10:*** Find the index of minimum error and select the best feature vector from the index (see Figure 7).

We applied INCA to generate the feature vector and obtain the best features for classification. INCA selected 340, 509, 735 and 101 features for DB1, DB2, DB3 and DB4 databases, respectively. The plots of misclassification rate versus the number of features obtained for various databases using Cubic SVM with 10-fold cross-validation are shown in Figure 7.

#### 2.2.3. Classification

The classification was performed using an SVM [33,34] classifier. The hyperparameters of the SVM were tuned using Bayesian optimisation to reach the optimum performance. The number of iterations of the Bayesian optimisation was chosen to be 30, and the fitness function was the misclassification rate. The hyperparameters of the SVM classifier (tabulated in Table 1) were fed as input to the Bayesian optimisation technique. The main purpose of using Bayesian optimisation was to obtain fine-tuned SVM, and the hyperparameter ranges used for Bayesian optimisation are given in Table 1.

The parameters of the used SVM classifiers for both databases are given in Table 2.

The validation technique of the given classifiers (see Table 2) was chosen as 10-fold cross-validation.

The last step of the COVID-19FclNet9 is classification, and these steps are given below.

***Step 11:*** Tune parameters of SVM using Bayesian optimisation.

***Step 12:*** Classify the selected optimal feature vector using fine-tuned SVM.

## 3. Results

This work used four X-ray image databases to validate the proposed Exemplar COVID-19FclNet9 model. A simple configured PC was used to obtain the results of this model. The system configurations of the used PC are as follows. It has an i9-9900 processor, 48 GB memory, 256 GB solid-state disk, and Windows 10.1 Professional operating system. In addition, MATLAB (2020b) has been utilised as a programming environment.

To evaluate the performance of the proposed model, four databases were used. Accuracy, precision, recall and F1 score metrics were employed to evaluate the performance of the developed model. The results obtained for the four databases are listed in Table 3, Table 4, Table 5 and Table 6.

The biggest database used was DB3, and its results were obtained with 10-fold cross-validation. The calculated confusion matrix of our proposed Exemplar COVID-19FclNet9 model using the DB3 database is denoted Table 5.

The overall results (accuracy, unweighted average recall, overall precision and overall F1 scores) obtained using our proposed model with four databases is shown in Table 7.

It can be noted from Table 7 that our proposed model obtained 97.60%, 89.96%, 98.84% and 99.64% accuracies using DB1, DB2, DB3 and DB4 databases, respectively.

**Figure 8:**
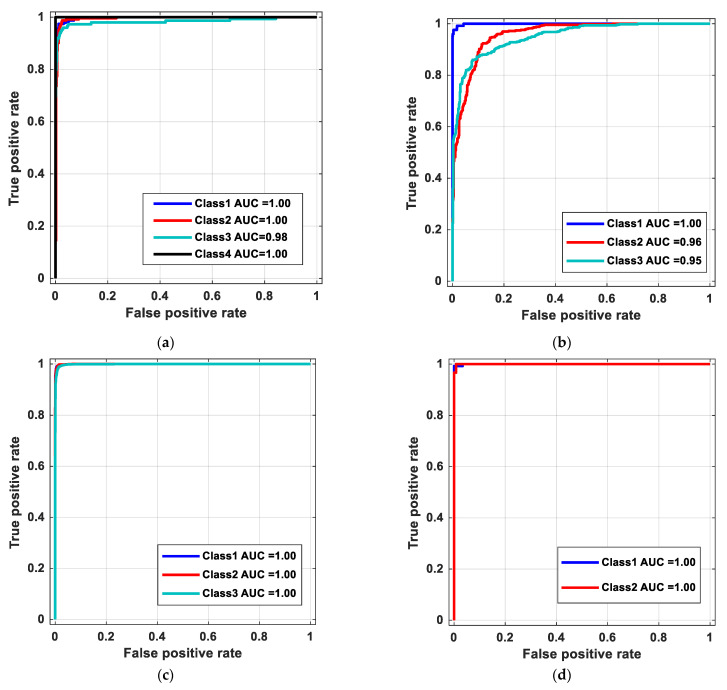
ROC curves of proposed model for datasets used: (**a**) DB1, (**b**) DB2, (**c**) DB3 and (**d**) DB4.

Moreover, ROC curves obtained using our proposed COVID-19FclNet9 model for various datasets used are denoted in Figure 8.

## 4. Discussion

Four X-ray image corpora were used in this work to validate the proposed Exemplar COVID-19FclNet9 model. It can be noted from our results that the developed model yielded high classification performance using all four databases. The proposed model is cognitive, and we used feature engineering and transfer learning to design this architecture. This model has three fundamental phases, and the most crucial phase of the proposed model is feature extraction. Our proposed deep feature generation model is a cognitive model and is also designed as a machine learning model. This feature extractor selects the most appropriate three feature vectors. It generates features using patches and original X-ray images, and these features are merged. NCA chooses the best 1000 features, which are classified using SVM classifier. The used pre-trained deep feature generation models are listed in Table 8.

**Figure 9:**
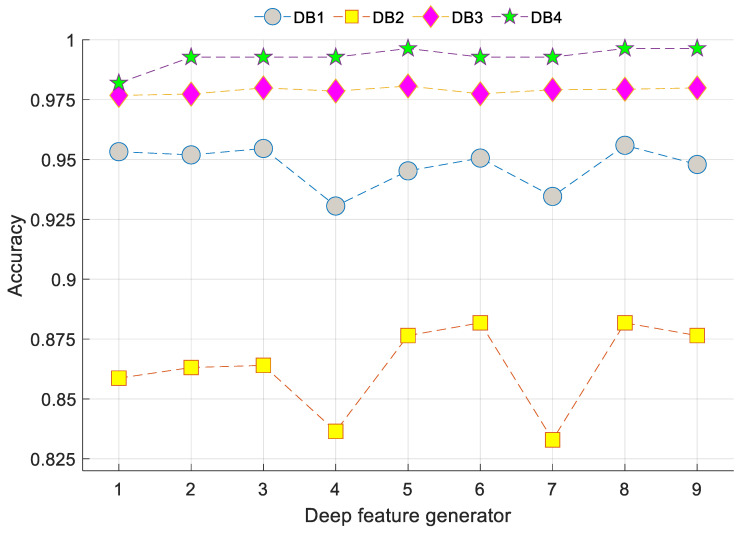
Graph of accuracies versus number of features used for various datasets used.

Table 8 shows the deep feature generation functions used in the Exemplar COVID-19FclNet9 model. The graph of accuracies versus number of features used with various databases is depicted in Figure 9.

Figure 9 shows that the range of accuracies calculated for DB1 varies from 93.06% (minimum accuracy) to 95.59% (maximum accuracy), and this range can be expressed as [93.06%, 95.59%]. Moreover, the obtained accuracy ranges for DB2, DB3 and DB4 were [83.29%, 88.18%], [97.68%, 98.07%] and [98.19%, 99.64%], respectively, using nine deep feature generation methods. The best three feature generators used for DB1 were the 8th (fc7 layer of the VGG19), 3rd (fc6 layer of the AlexNet) and 1st (fc8 layer of the AlexNet) deep feature generators. The selected three deep features for DB2 belonged to 6th (fc6 layer of the VGG16), 8th (fc7 layer of the VGG19) and 5th (fc7 layer of the VGG16) deep feature generators. The top three feature generators for DB3 were the 5th (fc7 layer of the VGG16), 3rd (fc6 layer of the AlexNet) and 9th (fc6 layer of the VGG19) transfer learning-based deep feature generators. Furthermore, 5th (fc7 layer of the VGG16), 8th (fc7 layer of the VGG19) and 9th (fc6 layer of the VGG19) were selected as the top three deep feature generators.

By merging these features and applying the INCA selector, the accuracy rates were increased from 95.59% to 97.60% for DB1, from 88.18% to 89.96% for DB2 and from 98.07% to 98.84% for DB3. Moreover, this strategy yielded the maximum performance with the minimum number of features (100 features) on the DB4.

The comparison of our work with other similar published works is shown in Table 9.

It can be noted from Table 9 that our proposed method has outperformed all the state-of-the-art techniques and is found to be robust as we have tested with four different databases. We used four X-ray image datasets to evaluate the presented COVID-19FclNet9. We used both small and large datasets. Murugan and Goel [54] applied a CNN to classify COVID-19, pneumonia and healthy classes. Their used dataset contained 2700 images, and each category had 900 images. Gilanie et al. [55] used a large X-ray image dataset, and their image dataset contained 15,108 X-ray images. They only applied VGG-16 network and reached 96% classification accuracy.

Ozturk et al. [48] proposed a convolutional neural network using an X-ray image dataset of three classes (COVID-19, pneumonia and healthy). Furthermore, their dataset is a heterogeneous dataset and was the DB2 dataset in our work. Ozturk et al. [48] achieved 87.02% accuracy using their model, while our COVID-19FclNet9 reached 89.96% accuracy using the same dataset. Hussain et al. [58] presented a CNN-based CoroDet model using three datasets and attained 99.10%, 94.20% and 91.20% classification accuracies, respectively. They did not use transfer learning; hence, the time complexity of their model may be higher than that for our proposal. Sitaula and Hossain [61] used transfer-learning-based deep X-ray image classification and used three image datasets. They reached 79.58%, 85.43% and 87.49% accuracies for three image datasets, respectively. Our COVID-19FclNet9 is a transfer-learning-based model, and it attained higher accuracies than that of the Sitaula and Hossain [61] transfer-learning-based classification model. Other methods in Table 5 used a CNN model with smaller X-ray image datasets. In this respect, our work is one of the first to use four datasets, as shown in Table 9, and obtained a higher classification performance for X-ray image classification. This justifies that our proposed model is accurate and robust.

The important salient features of the proposed Exemplar COVID-19FclNet9 are given below.

- A new deep feature generation architecture is presented using three pre-trained networks, and the proposed architecture can select the best feature generation model.
- This exemplar and cognitive deep feature generation model tested using four COVID-19 X-ray image databases and attained a high success rate on all databases, which justifies the universal success of this model.
- This model attained 97.60%, 89.96%, 98.84% and 99.64% accuracies using four databases (DB1, DB2, DB3 and DB4, respectively).
- Our method obtained the highest performance compared to other state-of-the-art works (see Table 9).
- The proposed method is a cognitive model because it can automatically select the best models, best features and most appropriate classifier.
- The proposed model yielded the highest classification performance using deep feature generators.
- The proposed model can detect COVID-19 and pneumonia accurately using X-ray images.

## 5. Conclusions

COVID-19 detection using medical images is a topic of immense interest in medical and healthcare research. Many methods have been proposed to detect COVID-19 accurately using image processing and machine learning techniques. For example, deep networks have been applied to COVID-19 cases using X-ray images. Our work here has presented a new Exemplar COVID-19FclNet9 framework to detect COVID-19 cases automatically. In this framework, nine deep feature extraction methods are obtained using three deep networks and this framework selects the most appropriate features. Using the proposed hybrid deep feature extractor, iterative feature selector and optimised SVM, a highly accurate model is then obtained. This model was tested on several X-ray image databases to confirm the universal classification. Vision transformers inspired this learning framework (Exemplar COVID-19FclNet9) and helped increase the performance of pre-trained deep feature extractors. Our proposed Exemplar COVID-19FclNet9 attained accuracies of 97.60%, 89.96%, 98.84% and 99.64% for four X-ray image databases (DB1, DB2, DB3 and DB4, respectively). It can be noted from these results that COVID-19FclNet9 is an effective computer vision model presented using AlexNet, VGG16 and VGG19 networks. In future work, variable pre-trained deep networks can be used in this architecture to improve performance.

## Figures and Tables

**Table 1:** Hyperparameter ranges tuned by Bayesian optimiser for SVM classifier.

Hyperparameter	Value
Multiclass method	One-vs.-One, One-vs.-All
Box constraint level	[0.001–1000]
Kernel	Cubic, Quadratic, Linear, Gaussian
Standardise	False, True

**Table 2:** Hyperparameters used for SVM classifiers with various databases.

Hyperparameter	Tuned Parameters for the DB1	Tuned Parameters for the DB2	Tuned Parameters for the DB3
Multiclass method	One-vs.-One	One-vs.-All	One-vs.-All
Kernel	Linear	Gaussian	Cubic
Box constraint	999.30	2	1
Standardise	False	True	True

**Table 3:** Results obtained using our proposed Exemplar COVID-19FclNet9 model with DB1 database.

Actual Class	Predicted Class			
	Normal	Bacterial Pneumonia	Virus Pneumonia	COVID-19
Normal	227	4	3	0
Bacterial Pneumonia	3	238	1	0
Viral Pneumonia	3	4	141	0
COVID-19	0	0	0	125
Recall (%)	97.01	98.35	95.27	100
Precision (%)	97.42	96.75	97.24	100
F1-score (%)	97.22	97.54	96.25	100

**Table 4:** Results obtained using our proposed Exemplar COVID-19FclNet9 model with DB2 database.

Actual Class	Predicted Class		
	COVID-19	Healthy	Pneumonia
COVID-19	120	0	5
Healthy	1	457	42
Pneumonia	0	65	432
Recall (%)	96	91.40	87
Precision (%)	99.17	87.55	90.25
F1-score (%)	97.56	89.43	88.59

**Table 5:** Results obtained using our proposed Exemplar COVID-19FclNet9 model with DB3 database.

Actual Class	Predicted Class		
	COVID-19	Pneumonia	Healthy
COVID-19	3586	2	28
Pneumonia	2	1318	25
Healthy	28	19	3953
Recall (%)	99.17	97.99	98.82
Precision (%)	99.17	98.43	98.68
F1-score (%)	99.17	98.21	98.75

**Table 6:** Results obtained using our proposed Exemplar COVID-19FclNet9 model with DB4 database.

Actual Class	Predicted Class	
	COVID-19	Healthy
COVID-19	126	1
Healthy	0	150
Recall (%)	99.21	100
Precision (%)	100	99.33
F1-score (%)	99.60	99.66

**Table 7:** Overall results (%) obtained using our proposed model using four databases.

Overall Results	DB1	DB2	DB3	DB4
Accuracy (%)	97.60	89.96	98.84	99.64
Unweighted average recall (%)	97.66	91.47	98.66	99.61
Precision (%)	97.85	92.32	98.76	99.80
F1 score (%)	97.75	91.86	98.71	99.63

**Table 8:** Deep feature generation functions used in the Exemplar COVID-19FclNet9 model.

Network	Number	Fully Connected Layer
AlexNet	1	fc8
	2	fc7
	3	fc6
VGG16	4	fc8
	5	fc7
	6	fc6
VGG19	7	fc8
	8	fc7
	9	fc6

**Table 9:** Comparison of our work with other similar published works.

Study	Method	Classifier	Split Ratio	Number of Class/Type	Number of Cases	Results (%)
Murugan and Goel [54]	Convolutional neural networks (ResNet50)	Softmax	70:30	3/Chest X-ray	900 COVID-19 900 Pneumonia 900 Normal	Acc: 94.07 Sen: 98.15 Spe: 91.48 Rec: 85.21 Pre: 98.15 F1: 91.22
Gilanie et al. [55]	Convolutional neural networks	Softmax	60:20:20	Chest radiology	1066 COVID-19 7021 Pneumonia 7021 Normal	Acc: 96.68 Spe: 95.65 Sen: 96.24
Pandit et al. [56]	Convolutional neural networks (VGG-16)	Softmax	70:30	1. 2/Chest radiographs 2. 3/Chest radiographs	1. 224 COVID-19 504 Healthy 2. 224 COVID-19 700 Pneumonia 504 Healthy	Acc: 1. 96.00 2. 92.53
Nigam et al. [57]	Convolutional neural networks (EfficientNet)	Softmax	70:20:10	3/Chest X-ray	795 COVID-19 795 Normal 711 Others	Acc: 93.48
Hussain et al. [58]	Convolutional neural networks (CoroDet)	Softmax	5-fold cross validation	1. 2/Chest X-ray 2. 3/Chest X-ray 3. 4/Chest X-ray	1. 500 COVID-19 800 Normal 2. 500 COVID-19 800 Normal 800 Pneumonia—bacterial 3. 500 COVID-19 800 Normal 400 Pneumonia—bacterial 400 Pneumonia—viral	Acc: 1. 99.10 2. 94.20 3. 91.20
Ozturk et al. [48]	Deep neural networks	Darknet-19	5-fold cross validation	3/Chest X-ray	125 COVID-19 500 Pneumonia 500 No Findings	Acc: 87.02 Sen: 92.18 Spe: 89.96
Shi et al. [59]	Deep neural networks	Deep neural networks	70:20:10	1. 3/Chest CT images 2. 3/Chest X-ray	1. 349 COVID-19 384 Normal 304 CAP 2. 450 COVID-19 1800 Normal 1837 CAP	Acc: 1. 87.98 2. 93.44
Mukherjee et al. [60]	Convolutional neural network, Deep neural network	Softmax	10-fold cross validation	2/Computed Tomography and Chest X-ray	336 COVID-19 336 non-COVID-19	Acc: 96.28 Sen: 97.92 Spe: 94.64 Pre: 94.81 F1: 96.34
Sitaula and Hossain [61]	Convolutional neural networks	FC-layers, and Softmax	70:30	1. 3/Chest X-ray 2. 4/Chest X-ray 3. 5/Chest X-ray	Database1: 125 COVID-19 125 No findings 125 Pneumonia Database2: 320 COVID-19 320 Normal 320 Pneumonia Bacterial 320 Pneumonia Viral Database3: 320 COVID-19 320 Normal 320 Pneumonia Bacterial 320 Pneumonia Viral 320 No findings	Acc: 1. 79.58 2. 85.43 3. 87.49
Our method	Exemplar COVID-19FclNet9	Support vector machine	10-fold cross validation	4/Chest X-ray	234 Control 242 Bacterial Pneumonias 148 Viral pneumonias 125 COVID-19	Acc: 97.60
				3/Chest X-ray	125 COVID-19 500 Pneumonia 500 Control	Acc: 89.96
				3/ Chest X-ray	3616 COVID-19 1345 Pneumonia 4000 Control	Acc: 98.84
				2/Chest X-ray	127 COVID-19 150 Normal	Acc: 99.64

## Data Availability

The data are not publicly available due to restrictions regarding the Ethical Committee Institution.
